# Microbial Abundances Retrieved from Sequencing data—automated NCBI Taxonomy (MARS): a pipeline to create relative microbial abundance data for the Microbiome Modelling Toolbox and utilizing homosynonyms for efficient mapping to resources

**DOI:** 10.1093/bioadv/vbae068

**Published:** 2024-05-10

**Authors:** Tim Hulshof, Bram Nap, Filippo Martinelli, Ines Thiele

**Affiliations:** School of Medicine, University of Galway, Galway H91 TK33, Ireland; Digital Metabolic Twin Centre, University of Galway, Galway H91 TK33, Ireland; School of Medicine, University of Galway, Galway H91 TK33, Ireland; Digital Metabolic Twin Centre, University of Galway, Galway H91 TK33, Ireland; School of Medicine, University of Galway, Galway H91 TK33, Ireland; Digital Metabolic Twin Centre, University of Galway, Galway H91 TK33, Ireland; School of Medicine, University of Galway, Galway H91 TK33, Ireland; Digital Metabolic Twin Centre, University of Galway, Galway H91 TK33, Ireland; Discipline of Microbiology, University of Galway, Galway H91 TK33, Ireland; Ryan Institute, University of Galway, Galway H91 TK33, Ireland; APC Microbiome Ireland, Cork T12 K8AF, Ireland

## Abstract

**Motivation:**

Computational approaches to the functional characterization of the microbiome, such as the Microbiome Modelling Toolbox, require precise information on microbial composition and relative abundances. However, challenges arise from homosynonyms—different names referring to the same taxon, which can hinder the mapping process and lead to missed species mapping when using microbial metabolic reconstruction resources, such as AGORA and APOLLO.

**Results:**

We introduce the integrated MARS pipeline, a user-friendly Python-based solution that addresses these challenges. MARS automates the extraction of relative abundances from metagenomic reads, maps species and genera onto microbial metabolic reconstructions, and accounts for alternative taxonomic names. It normalizes microbial reads, provides an optional cut-off for low-abundance taxa, and produces relative abundance tables apt for integration with the Microbiome Modelling Toolbox. A sub-component of the pipeline automates the task of identifying homosynonyms, leveraging web scraping to find taxonomic IDs of given species, searching NCBI for alternative names, and cross-reference them with microbial reconstruction resources. Taken together, MARS streamlines the entire process from processed metagenomic reads to relative abundance, thereby significantly reducing time and effort when working with microbiome data.

**Availability and implementation:**

MARS is implemented in Python. It can be found as an interactive application here: https://mars-pipeline.streamlit.app/along with a detailed documentation here: https://github.com/ThieleLab/mars-pipeline.

## 1 Introduction

The study of the microbiome has rapidly gained importance due to its significant influence on human health and its potential to drive advances in areas, such as therapeutics, nutrition, and environmental sciences (Hou *et al.* 2022). Metagenomic sequencing has provided researchers with an unprecedented ability to characterize the composition and function of microbial communities within the microbiome.

Functional characterization is increasingly done using computational approaches with constraint-based metabolic modelling being a prominent method (Basile *et al.* 2021, Beura *et al.* 2022). This approach requires microbial metabolic reconstructions from resources, such as AGORA2 (Heinken *et al.* 2023a) and APOLLO (Heinken *et al.* 2023b). Using the Microbiome Modelling Toolbox (Baldini *et al.* 2019), which is part of the COBRA Toolbox v3 (Heirendt *et al.* 2019), tens or hundreds of these microbial metabolic reconstructions can be combined into a metagenomic sample-specific microbiome-level metabolic model. Subsequently, *in silico* metabolic traits of the *in silico* microbial community can be computed using, e.g. flux balance analysis (Orth *et al.* 2010). Yet, preprocessing 16S rRNA or shotgun metagenomic data for the generation of microbiome-level metabolic models remains a manual, laborious, and prone to errors effort. Additionally, the presence of homosynonyms, i.e. alternative taxonomic names, complicates the task of identifying species within the resources.

Here, we present MARS, a Python-based extension to the Microbiome Modelling Toolbox. MARS (i) automates the identification of homosynonyms, (ii) extracts relative abundances, (iii) maps species and genera onto a reconstruction resource of the user’s choice. Furthermore, (iv) MARS normalizes microbial reads, introduces an optional cutoff, and ensures the resultant relative abundance table is seamlessly integrated with the Microbiome Modelling Toolbox. Finally, (v) MARS computes the α-diversity before and after mapping, enabling an assessment of the correlation between initial microbial reads and final microbial relative abundances. When provided with stratification information, the pipeline can also perform basic statistical analyses to elucidate differences between groups.

## 2 Methods

MARS was implemented in Python and uses the pandas library (Pandas Development Team 2020) for all data manipulation. MARS can be called from the Microbiome Modelling Toolbox, implemented in Matlab (see Github installation process).

### 2.1 Input data and preprocessing

The input to MARS consists of what we refer to as ‘QIIME2 format’ (Bolyen *et al.* 2019), or similarly prepared tables: a taxonomy table and a feature table (see Tables 1 and 2 for an example) or the already merged version of these two (Table 3). It is important to clarify that both 16S rRNA and shotgun metagenomic sequencing data are supported, as long as they are presented in the required format. The taxonomy table lists the taxonomic information for the detected microorganisms in the samples, while the feature table provides the observed abundance of each microbe in each sample. Both tables share a common index column, which links each row in the feature table to its corresponding taxonomic identification in the taxonomy table. MARS merges these two tables into a single dataset. Note that MARS omits the ‘confidence’ column, which indicates the reliability of the taxonomic assignment, present in the taxonomy table.

**Table 1.** Feature table example: this table shows an example of the feature table input for MARS, specifying OTU abundances in different samples.

OTU ID	ERS941248	ERS1265423	ERS940989
4b5e	6162.0	0.0	4978.0
f319	5119.0	0.0	0.0

ERS941248, ERS1265423, ERS940989 are sample IDs.

**Table 2.** Taxonomy table example: this table shows an example of the taxonomy table input for MARS, specifying the taxonomy and confidence levels for different feature IDs.

Feature ID	Taxon	Confidence
4b5e	k__Bacteria; p__Bacteroidetes; c__Bacteroidia; o__Bacteroidales; f__Bacteroidaceae; g__Bacteroides; s__	0.958
f319	k__Bacteria; p__Bacteroidetes; c__Bacteroidia; o__Bacteroidales; f__Prevotellaceae; g__Prevotella; s__copri	0.995

k, kingdom; p, phylum; c, class; o, order; g, genus; s, species.

**Table 3.** Taxonomy table example: this table shows an example of an already merged table.

Taxon	ERS941248	ERS1265423	ERS940989
k__Bacteria; p__Bacteroidetes; c__Bacteroidia; o__Bacteroidales; f__Bacteroidaceae; g__Bacteroides; s__	6162.0	0.0	4978.0
k__Bacteria; p__Bacteroidetes; c__Bacteroidia; o__Bacteroidales; f__Prevotellaceae; g__Prevotella; s__copri	5119.0	0.0	0.0

### 2.2 Splitting into taxonomic groups

The dataset then undergoes further refinement to segregate data based on taxonomic classification, namely kingdom, phylum, class, order, family, genus, and species. Each taxon name at each taxonomic level is associated with the corresponding abundance to create distinct taxonomic level-specific datasets, deemed dataframes.

### 2.3 Acquiring taxonomy NCBI IDs and scraping alternative species names

MARS then addresses the issue of alternative taxonomic names, spellings, and homosynonyms by cross-referencing microbial taxa with predetermined alteration patterns, specific alterations, and homosynonyms derived from a user-chosen resource. Alternatively, MARS can obtain the taxonomy ID for each species using the Entrez tool in Biopython module (Cock *et al.* 2009). Therefore, the user uploads a file containing a list of species names, e.g. the output from section Splitting into taxonomic groups. MARS uses these names to query the NCBI database via the Entrez.esearch function. The function returns an ID list, from which the taxonomy ID is extracted. Once the taxonomy ID is obtained, MARS uses this identifier to automate a search on the NCBI Taxonomy Browser. The tool can access the specific page of each species using its taxonomy ID and obtain all alternative names (homosynonyms) listed. This is achieved through web scraping techniques using the Python library requests_html.

### 2.4 Mapping of the dataframes onto the microbial reconstruction resource

MARS takes the collected species names and alternative names and checks their presence in a user’s defined resource, such as the AGORA2 resource (default). It compares the names with those listed, identifying matches and subsequently mapping the species to the resource. The results of this process are species that exist in the chosen resource, and species that have an alternative name present in the resource. This step ensures maximum coverage and accurate mapping of dataset onto the resource.

### 2.5 Data normalization

For each taxonomic level, MARS computes the sum of abundances for each unique taxon present in the dataframes. If a cutoff value is provided, then MARS removes low-abundance taxa, whose summed values are below the specified cutoff. The abundance data in each column (i.e. sample) are scaled such that the sum of all values in the column is equal to 1.

### 2.6 Microbial community metrics

MARS determines several metrics for each taxonomic level to analyse the microbial communities for the (i) input dataset, (ii) processed dataframes (i.e. only microbes that are present in the resource), and (iii) input data that could not be mapped onto the resource (i.e. microbes that are absent from the resource). The metrics include α-diversity, read counts, and, at the phylum level, the Firmicutes to Bacteroidetes ratio and are calculated as follows.

#### 2.6.1 α-diversity

The α-diversity representing the diversity within a sample is computed using the Shannon index (*H*):

(1) $$H=-\sum_{i=1}^{S}\left(p_{i}\cdot \ln(p_{i})\right)$$

where *S* is the total number of taxa in the sample, $p_{i}$ is the proportion of individuals belonging to the *i*-th species in the dataframe, and ln is the natural logarithm.

#### 2.6.2 Read counts

The total read counts are computed by summing the values representing the total abundance of each taxon across all samples in each dataframe.

#### 2.6.3 Firmicutes to bacteroidetes ratio

The ratio is calculated by first grouping the data by phylum and summing the read counts. It then retrieves the sum of the read counts for the Firmicutes and Bacteroidetes and calculates the ratio by dividing the sum of read counts for Firmicutes by that for Bacteroidetes. In cases where one of these phyla is not presented in the data, a value of 0 is used in the calculation to avoid division by zero errors.

### 2.7 Statistical analysis of stratified data

When stratification information is provided, MARS can perform basic statistical analyses on the processed data. Metrics, such as α-diversity, read count, resource mapping, and Firmicutes/Bacteroidetes ratio, can be assessed for significant differences between groups.

### 2.8 Output

Data are saved into four categories: ‘normalized’, ‘present’, ‘absent’, and ‘metrics’. Each category contains relevant tables, which are saved to the user’s desired file format (i.e. csv, txt, excel, json, and parquet) for subsequent analyses, e.g. input for the Microbiome Modelling Toolbox (Baldini *et al.* 2019), and as a reference.

## 3 Discussion

MARS offers a comprehensive solution to the challenges researchers face when processing and analysing microbial abundances in 16S rRNA and shotgun metagenomic datasets. Designed to seamlessly integrate with the Microbiome Modelling Toolbox (Baldini *et al.* 2019), it automates the previously manual process of converting raw data into relative abundance information and its subsequent mapping onto the AGORA2 resource. With option of MARS to obtain automatically NCBI taxonomies, MARS addresses the specific challenge of identifying species homosynonyms. This functionality streamlines the process of taxonomy ID acquisition from NCBI and the subsequent scraping of homosynonyms. The automation not only saves invaluable time but also enhances the accuracy and completeness of the results by ensuring a thorough collection of homosynonyms. Beyond its primary function, MARS facilitates a deeper understanding of microbial datasets by extracting vital metrics and enabling comparisons among various groups within the data. By providing a standardized pipeline, MARS fosters reproducibility and consistency in *in silico* microbiome research, ensuring uniform data processing and analysis across different projects.

However, MARS does have its limitations. Its inability to handle raw metagenomic sequencing data directly means that researchers need to use external tools, such as QIIME2 (Bolyen *et al.* 2019), for initial data processing. This dependency creates potential format disparities, as the current version of MARS primarily supports QIIME2’s output format. While this reliance limits MARS’ versatility, researchers can still utilize MARS by converting alternative file formats to the one MARS recognizes, as illustrated in Tables 1–3. MARS reliance on the NCBI Taxonomy database is both its strength and potential weakness. The accuracy and comprehensiveness of the taxonomy mapping results are directly tied to the quality of NCBI’s data. Future iterations of MARS could potentially benefit from mechanisms that cross-reference with other taxonomic databases, bolstering its reliability and extending its utility.

## 4 Conclusion

MARS is a valuable tool for researchers by addressing the important challenge of taxonomic name identification and mapping, and by streamlining data processing and analysis and allowing them to delve deeper into the biological revelations of their datasets. By automating and expediting this process, MARS further empowers researchers to shift their focus towards the functional analysis of microbiome data.
